# Noninvasive biventricular pressure-strain-volume loop-derived myocardial work analysis in competitive athletes

**DOI:** 10.1038/s41598-026-35206-0

**Published:** 2026-01-08

**Authors:** Andrea Ferencz, Ádám Szijártó, Tímea Katalin Turschl, Zsuzsanna Ladányi, Bálint Károly Lakatos, Márton Tokodi, Máté Tolvaj, Márk Zámodics, Máté Babity, Regina Benkő, Orsolya Kiss, Emese Csulak, Nóra Sydó, Csongor Meskó, Hajnalka Vágó, Béla Merkely, Attila Kovács, Alexandra Fábián

**Affiliations:** 1 Heart and Vascular Center, Semmelweis University, 68 Városmajor Street, Budapest, 1122 Hungary; 2 Argus Cognitive, Inc, Hanover, NH USA; 3 Department of Experimental Cardiology and Surgical Techniques, Semmelweis University, Budapest, Hungary; 4 Department of Sports Medicine, Semmelweis University, Budapest, Hungary

**Keywords:** 3D echocardiography, Athlete’s heart, Myocardial work, Pressure-strain-volume loop analysis, Right ventricle, Exercise capacity, Cardiology, Echocardiography

## Abstract

Intense exercise imposes hemodynamic load on the heart, and while morphological remodeling is well-characterized, assessment of exercise-induced functional changes, like enhanced contractility, remains challenging. We aimed to introduce a novel 3D echocardiography (3DE)-derived method for noninvasive quantification of biventricular systolic function, less dependent on loading conditions in competitive athletes, and to explore the relationship with peak exercise capacity. We enrolled 260 athletes and 24 sedentary volunteers. All subjects underwent 3DE to measure left (LV) and right ventricular (RV) volumes and ejection fractions (EF). Biventricular global longitudinal strain (GLS) tracings and noninvasively estimated pressure curves were concatenated and further adjusted to instantaneous volumes to create pressure-strain-volume loops and derive volume-adjusted myocardial work (MW) indices (LV GWIV and RV GWIV). Athletes had lower biventricular EF and LV GLS, but significantly higher LV GWIV (10273 ± 2929 vs. 7387 ± 2050 mmHg%·mL, *p* < 0.001) and RV GWIV (3422 ± 1339 vs. 2436 ± 796 mmHg%·mL, *p* < 0.001) compared to controls. Among the functional echocardiographic parameters, RV GWIV showed the strongest correlation (*r* = 0.30, *p* < 0.001) and was an independent predictor of exercise capacity. Our novel metrics captured enhanced biventricular function in athletes at rest, and RV GWIV was independently associated with higher peak exercise capacity.

**Supplementary Information:**

The online version contains supplementary material available at 10.1038/s41598-026-35206-0.

## Introduction

The impact of regular physical training on cardiac chambers has been a recurrent subject of research in recent decades^1^. Intense exercise demands an increased cardiac output, leading to functional and morphological adaptations in the athlete’s heart, permitting the higher hemodynamic demands during physical activity^2–4^. These adaptive changes typically include increased myocardial mass, dilatation of the heart chambers, lowered resting heart rate, enhanced myocardial contractility, and thus, cardiac performance^2,5,6^, which ultimately enables athletes to perform at a higher exercise capacity^1,7^, features previously recognized as specific to endurance athletes.

In clinical practice, echocardiography is the most commonly used imaging modality to assess exercise-induced cardiac adaptation. Echocardiographic metrics such as ejection fraction (EF) and global longitudinal strain (GLS) are routinely used to assess systolic function in athletes. Nevertheless, these measures are significantly influenced not only by age, sex, training regimes, or types of sports but also by ventricular dilation and heart rate, along with the corresponding loading conditions. Preload and afterload are often considerably altered in athletes due to enhanced blood volume and lower systemic vascular resistance^3,5,6,8^. Consequently, although athletes demonstrate an increased contractile reserve, resting EF and GLS values often remain within the low-normal range^9–11^. Although it is a well-established pattern in this population, it still contributes to the challenge of interpreting conventional echocardiographic parameters at rest.

Advanced imaging techniques, such as three-dimensional echocardiography (3DE) and myocardial deformation imaging, have gained attention in recent years as additions to conventional diagnostic methods^5^. Pressure-strain loop-derived myocardial work (MW) metrics allow the noninvasive assessment of subtle changes in systolic function, less dependent on afterload^12,13^. Still, a major limitation of these parameters is their substantial dependence on variations in preload and ventricular geometry, both of which play a fundamental role in volume-overload states, such as rigorous exercise^12,14^ hindering their utility as reliable markers of cardiac performance.

The gold-standard markers of intrinsic cardiac contractility measured by invasive pressure-volume (PV) analysis are distinguished for their independence of preload and afterload. However, the invasive nature of the examinations hampers their use in everyday clinical practice and athletic screening, and their utilization is almost exclusively limited to experimental settings. Thus, the need for a noninvasive, clinically applicable surrogate of contractility in athletes remains unmet. Of note, in a recently published work, our research group demonstrated that a marker incorporating instantaneous left ventricular (LV) size into the pressure-strain relationship correlates more closely to invasively measured LV contractility, thus reflects cardiac performance more adequately compared to conventional functional measures in a rat model of volume overload-induced LV dysfunction^12^. Therefore, integrating preload into the pressure-strain relationship may offer a physiologically more comprehensive approach that may capture the enhanced contractile reserve in athletes that conventional markers may not.

Accordingly, our study aimed to introduce a novel 3DE-derived method that incorporates preload, afterload, and ventricular geometry to noninvasively capture enhanced contractility even during resting conditions in a large cohort of competitive athletes. Additionally, we aimed to investigate the correlation between our novel parameters and peak exercise capacity.

## Methods

### Study design, patient selection and ethical approval

We retrospectively enrolled 260 healthy competitive athletes from our center’s comprehensive sports cardiology screening database. The cohort included individuals from endurance, power, and mixed sports disciplines, reflecting the subtly different cardiac adaptations associated with the distinct hemodynamic and mechanical demands of each sports discipline. Inclusion criteria were: i, available full volume 3D echocardiographic datasets for the analysis of both the LV and right ventricle (RV), ii, availability of cuff-based systemic blood pressure measurement to allow noninvasive LV pressure curve reconstruction, and iii, at least trace tricuspid regurgitation to allow non-invasive RV pressure curve reconstruction. Exclusion criteria were: i, the presence of any suspected or subsequently established cardiac pathology (such as hypertrophic cardiomyopathy, arrhythmogenic right ventricular dysplasia, valvular heart disease, or ischemic heart disease, etc.), ii, unavailability of CPET, and iii, insufficient 3DE image quality for further analysis. Detailed information on each athlete’s training regimen, medical background, and results from a standard physical examination - including brachial artery cuff blood pressure measurements and a 12-lead electrocardiogram (ECG) - was collected. Blood pressure was measured in both arms with the participant in a supine position following a 20-minute rest before the acquisition of the 3D echocardiographic images. All participants underwent cardiopulmonary exercise testing (CPET) to assess peak oxygen uptake (VO₂ and VO₂/kg), followed by comprehensive two-dimensional (2D) and 3DE. A control group of age- and sex-matched healthy individuals (*n* = 24) with sedentary lifestyles (engaging in less than 3 hours of physical activity per week and with no history of professional sports training) was also selected retrospectively. These individuals underwent the same screening protocol, including CPET.

Written informed consent was obtained from all participants. The study was carried out in accordance with the Declaration of Helsinki and approved by the Medical Research Council (ETT-TUKEB No. 13687-0/2011-EKU).

### Cardiopulmonary exercise testing

CPET for peak oxygen uptake (VO₂ and VO₂/kg) calculation was performed on a treadmill (T-2100, GE Healthcare, Finland) using our institution’s sport-specific incremental protocols. The test began with a brief resting phase (1 min sitting), followed by a 1–2 min warm-up walk at 6 km/h on a flat surface. Thereafter, subjects performed continuous uphill running starting at 8–10 km/h, with the slope increased by 1.0–1.5% each minute until exhaustion. Maximal effort was defined by subjective exhaustion in combination with physiological criteria, including a respiratory exchange ratio (RER) > 1.1, flattening of oxygen uptake or heart rate curves, and/or elevated blood lactate concentrations. To ensure maximal exertion, participants were strongly encouraged throughout the test. After termination, active recovery included 1 min of walking at 4 km/h followed by 4 min of rest. Expired gases were measured breath by breath using an automated cardiopulmonary exercise system (Respiratory Ergostik; Geratherm, Bad Kissingen, Germany), and all data were averaged over 10-second intervals. Reference values for non-athletes were provided by the manufacturer and adjusted for sex, age, height, and weight. Continuous ECG monitoring was performed (CAM-14 module, GE Healthcare, Finland). Blood lactate levels were sampled at rest, every 2 min during exercise, at peak load, and in the fifth minute of recovery (Laktate Scout 4+, EKF Diagnostik, Germany).

### 2D and 3D echocardiography

Transthoracic echocardiography acquisitions were obtained using commercially available ultrasound systems (Vivid E95, 4Vc-D probe, GE Vingmed Ultrasound, Horten, Norway, and EPIQ7, X5-1 probe, Philips Medical System, Best, The Netherlands). A standard acquisition protocol consisting of 2D loops from parasternal, apical, and subxiphoid views was applied. LV end-diastolic (LVIDd) and end-systolic internal diameters (LVIDs), and wall thicknesses were evaluated in the parasternal long-axis view. LV mass (Mi) indexed to body surface area (BSA) was estimated using the Devereux-formula. Left atrial (LA) 2D end-systolic volume index (LAVi) was assessed using the Simpson method indexed to BSA. LV inflow by pulsed-wave Doppler at the level of the mitral valve coaptation was obtained to determine early (E) and late (A) diastolic peak velocities, their ratio (E/A), and the E-wave deceleration time (DT). Pulsed-wave Doppler tissue imaging was used to measure systolic (s’), early (e’), and late (a’) diastolic peak velocities along with average E/e’ at the mitral lateral and medial annulus. Furthermore, RV basal short-axis diameter (RVd), tricuspid annular plane systolic excursion (TAPSE), fractional area change (FAC), RV free wall longitudinal strain (RVFWLS), RV septal longitudinal strain (RVSLS), pulmonary artery systolic pressure (PASP), and right atrial (RA) 2D end-systolic volumes (RAVi) were determined according to current guidelines^15^.

Beyond the conventional echocardiographic protocol, ECG-gated full-volume 3D datasets, reconstructed from four cardiac cycles optimized for LV or RV, respectively, were obtained from an apical view for further offline analysis. Three-dimensional datasets focused on the LV were processed using semi-automated, commercially available, thoroughly validated software (4D LV-Analysis, TomTec Imaging, Unterschleissheim, Germany). 3D LV end-diastolic volume index (EDVi), 3D end-systolic volume index (ESVi), 3D stroke volume index (SVi), and 3D LV mass index (LV Mi) were measured. For the assessment of global LV function, EF and 3D GLS were calculated. For the measurement of 3D RV EDVi, RV ESVi, RVEF, and RV GLS, the ReVISION software package (Argus Cognitive, Inc., Hanover, NH, USA) was used as previously described in detail^16^. Concerning 3DE-derived metrics, good interobserver and intraobserver variabilities were previously reported^16^.

### Three-dimensional myocardial work analysis

**Fig. 1:**
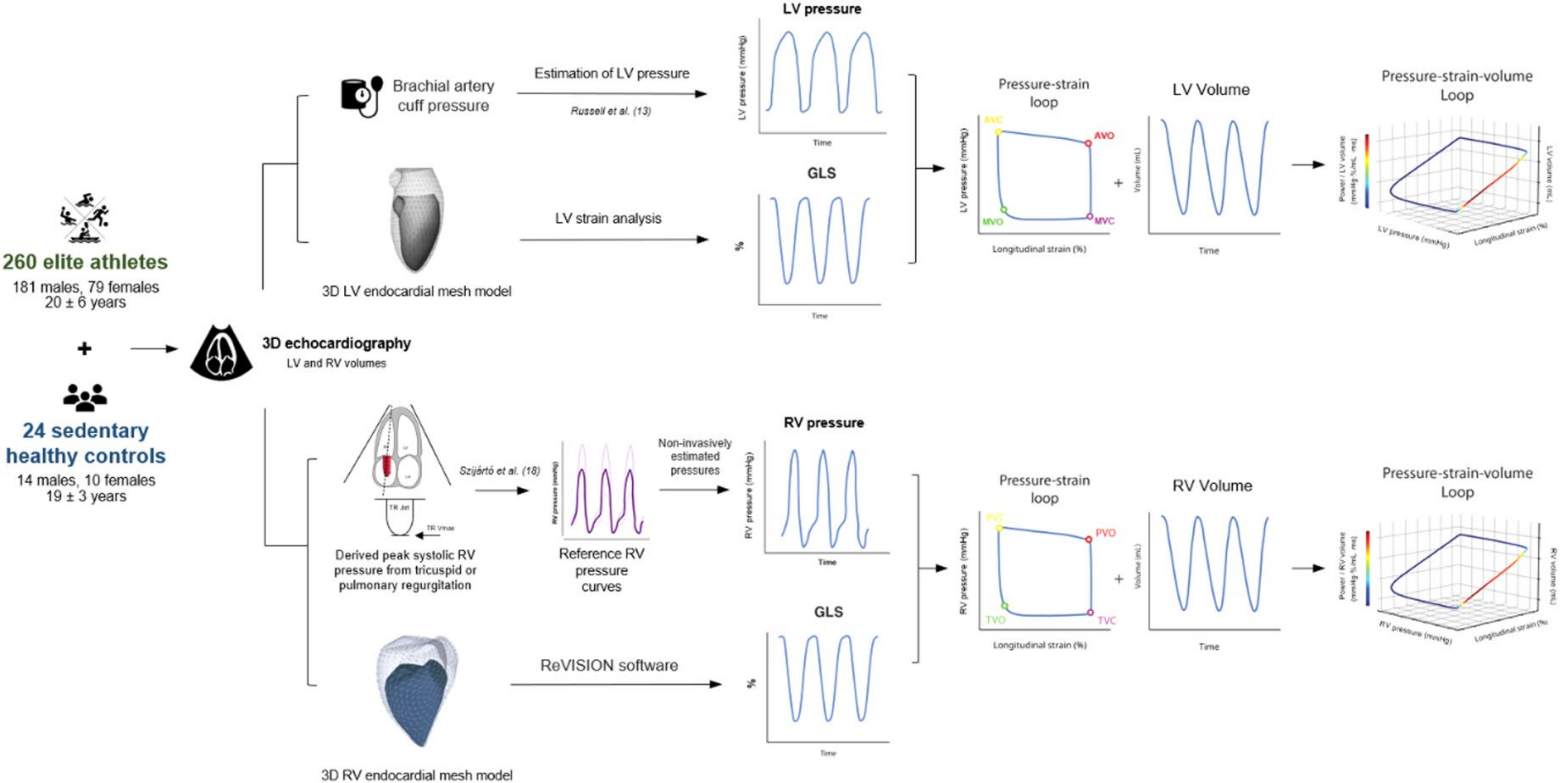
Schematic overview of the methodology used to assess conventional and volume-adjusted myocardial work parameters. 3D, three-dimensional; GLS, global longitudinal strain; LV, left ventricle; RV, right ventricle; TR, tricuspid regurgitation.

For LV MW analysis, 3D LV volumetric and GLS curves were exported from the commercially available software (TomTec). To estimate noninvasive LV pressure curves, brachial artery cuff pressure measurements were used following the principles outlined and published by Russell and colleagues^13,17^. Valvular event timings were tagged manually on the GLS curve by expert readers using the corresponding 3D image. Using a custom-made software, the estimated LV pressure and strain curves were concatenated in order to obtain 3D LV PS loops. Then, the 3D LV volumetric curves were fused into the PS relationship to generate LV pressure-strain-volume (PSV) loops. Similarly, for RV MW analysis, 3D RV volumetric and GLS curves were generated using the ReVISION software (Argus Cognitive). Noninvasive RV pressure curves were created using the tricuspid regurgitation peak velocity using an artificial intelligence-based tool, as described in detail^18^. The RV PSV-loops were created similarly to the left counterpart as explained above. The schematic overview of the methodology used to assess conventional and volume-adjusted MW parameters is outlined in Fig. 1.

Using LV and RV PS-loops, 3D MW indices were calculated as follows, global MW index (GWI), constructive MW index (GCW), wasted MW index (GWW) and MW efficiency (GWE), where GWI represents the amount of MW performed by the ventricles during systole and was computed by integrating the instantaneous power (the product of strain rate and pressure) from mitral or tricuspid valve closure to valve opening, GCW refers to the MW that contributes to effective ejection and filling, characterized by myocardial shortening during systole and lengthening during isovolumetric relaxation, GWW represents the ineffective work referring to myocardial lengthening during systole and shortening during isovolumetric relaxation, and GWE referring to MW efficiency calculated as GCW/ (GCW + GWW)^6,13,19,20^. Then, using PSV-loop analysis, the above-described MW parameters were adjusted to instantaneous 3D LV and RV volumes; thus, volume-adjusted global MW (GWIV), volume-adjusted constructive MW (GCWV), volume-adjusted wasted MW (GWWV), and volume-adjusted MW efficiency (GWEV) were calculated.

### Statistical analysis

Statistical analyses were conducted using a dedicated software (StatSoft Statistica, version 12, Tulsa, OK, USA). Continuous variables are presented as means ± standard deviation (SD), while categorical variables are presented as counts and percentages. To assess the normality of data distribution, we utilized the Shapiro-Wilk test. Group comparisons for continuous variables were performed using either the unpaired Student’s t-test or the Mann-Whitney *U* test, depending on data distribution. Categorical variables were compared using the chi-square (*χ²*) test or Fisher’s exact test, as appropriate. Multiple group comparisons were performed using ANOVA with Fisher’s LSD post-hoc test. Correlations between variables were examined using Pearson or Spearman correlation coefficients. To identify the independent predictors of VO₂/kg in the athlete cohort, multiple linear regression analysis was performed, with collinearity being assessed using the variance inflation factor (VIF), where values greater than 3 indicated multicollinearity. A *p*-value < 0.05 was considered statistically significant.

## Results

### Demographic, hemodynamic, and training-specific characteristics

Basic demographic, hemodynamic, and training-specific characteristics of the athlete and control groups are outlined in Table 1. When comparing the two groups, athletes presented with higher height, weight, and BSA. Additionally, athletes also exhibited higher resting systolic blood pressure (SBP), along with lower heart rates (HR), compared to the control group. The athletes have been participating in competitive sports for an average of 12 years and trained for a mean duration of 16 h/week at the time of the echocardiographic assessment. The athlete’s peak exercise capacity was significantly greater than that of the control population. The majority of athletes in our cohort participated in mixed (66.5%) and endurance sports (24.2%), with water polo, soccer, and swimming being the most common. Other sports types, including power and skill-based disciplines, were also represented (Table 1).

**Table 1:** Baseline and training-specific characteristics of athlete and control groups.

	Athletes (*n* = 260)	Controls (*n* = 24)	*p*
Baseline characteristics			
Age (years)	20.0 ± 5.7	19.0 ± 3.4	0.404
Male, *n* (%)	181 (69.6)	14 (58.3)	0.362
Height (cm)	177.5 ± 10.6	169.3 ± 11.2	0.003
Weight (kg)	71.7 ± 14.0	62.0 ± 11.8	0.001
BSA (m^2^)	1.8 ± 0.2	1.7 ± 0.1	0.004
SBP (mmHg)	131.5 ± 14.4	119.5 ± 12.0	< 0.001
DBP (mmHg)	74.3 ± 9.3	77.2 ± 11.0	0.150
HR (b.p.m.)	67.2 ± 12.5	74.5 ± 14.5	0.007
**Training-specific characteristics**			
Competitive training since (years)	12.0 ± 5.7	-	
Training time (h/week)	15.6 ± 7.3	-	
VO_2_ (L/min)	3.8 ± 0.9	2.8 ± 0.6	< 0.001
VO_2_/kg (mL/kg/min)	53.5 ± 7.6	45.7 ± 7.3	< 0.001
**Type of sport**			
Mixed, *n* (%)	173 (66.5)	-	
Endurance, *n* (%)	63 (24.2)	-	
Power, *n* (%)	19 (7.3)	-	
Skill, *n* (%)	5 (1.9)	-	

Continuous variables are presented as means ± SD; categorical variables are reported as frequencies (%). BSA, body surface area; DBP, diastolic blood pressure; HR, heart rate; SBP, systolic blood pressure; VO2, peak oxygen uptake; VO2/kg, peak oxygen uptake indexed to body weight.

Furthermore, we compared male (*n* = 181) and female (*n* = 79) athletes based on training-specific characteristics, with results summarized in Supplementary Table 1. There was no substantial difference in age between the two groups. Although male athletes participated in competitive sports activities for longer periods, the female athletes had longer average weekly training periods. Male athletes showed increased values in CPET-derived peak exercise capacity compared to female athletes.

We subsequently analyzed the variations between adolescent (< 18 years of age, *n* = 133) and adult (≥ 18 years of age, *n* = 127) athletes based on training-specific characteristics (Supplementary Table 2). Adolescent athletes had an average training duration of 13 h/week, whereas adult athletes trained for an average of 19 h/week. Despite the differences in the training regimes of the two age groups, adolescent athletes significantly exceeded the adult population regarding CPET-derived peak exercise capacity.

### 2D and 3D echocardiographic and advanced myocardial work analysis of biventricular morphology and function

We evaluated 2DE parameters in both athletes and controls, as detailed in Supplementary Table 3. The results demonstrated the well-established cardiac adaptations seen in athletes, reflected in the morphological and functional measures of both the LV and RV. However, no significant differences were observed between the groups in terms of TAPSE, PASP, and RVSLS. The 2DE parameters further showed significantly larger LV and RV dimensions of the male athletes compared to females, while RV functional parameters were higher among female athletes (Supplementary Table 4). Moreover, adult athletes showed larger LV dimensions while exhibiting reduced RVSLS compared to adolescent athletes (Supplementary Table 5).

The 3D morphological and functional echocardiographic parameters of athletes and controls are shown in Table 2. Regarding the LV, athletes had notably increased LV EDVi, ESVi, SVi, and Mi values compared to controls. However, athletes exhibited substantially lower resting values of LV EF, LV GLS. Similarly, athletes had greater RV dimensions in both systole and diastole (RV EDVi and ESVi), as well as RV SVi values in comparison to controls with lower resting RV EF values in athletes. We further performed advanced MW analysis, and while both LV and RV conventional MW parameters, such as GWI, GCW, GWW and GWE did not show any significant difference between athletes and controls, our novel LV and RV volume-adjusted global MW (GWIV) and volume-adjusted constructive MW (GCWV) parameters were considerably higher among athletes in comparison to controls. In addition, RV volume-adjusted wasted MW (GWWV) also exhibited greater values in athletes compared to controls (Table 2).

**Table 2:** 3D echocardiographic and biventricular myocardial work parameters of athlete and control groups.

	Athletes (*n* = 260)	Controls (*n* = 24)	*p*
Left ventricle			
LV EDVi (mL/m^2^)	82.6 ± 13.1	62.7 ± 9.7	< 0.001
LV ESVi (mL/m^2^)	35.9 ± 7.4	23.8 ± 4.5	< 0.001
LV SVi (mL/m^2^)	46.6 ± 7.4	38.8 ± 6.5	< 0.001
LV Mi (g/m^2^)	87.6 ± 15.1	67.3 ± 10.7	< 0.001
LV EF (%)	56.5 ± 4.2	59.9 ± 4.6	< 0.001
LV GLS (%)	-19.1 ± 2.2	-21.1 ± 2.3	< 0.001
**Right ventricle**			
RV EDVi (mL/m2)	83.3 ± 13.9	67.2 ± 11.5	< 0.001
RV ESVi (mL/m2)	37.6 ± 8.5	27.0 ± 5.9	< 0.001
RV SVi (mL/m2)	45.7 ± 7.1	40.2 ± 6.9	< 0.001
RV EF (%)	55.1 ± 4.7	59.9 ± 4.7	< 0.001
RV GLS (%)	-21.7 ± 3.4	-23.0 ± 4.1	0.070
**Left ventricular myocardial work**			
GWI (mmHg·%)	1975.4 ± 338.5	1991.6 ± 388.8	0.824
GCW (mmHg·%)	2012.6 ± 349.7	2027.0 ± 418.9	0.849
GWW (mmHg·%)	55.7 ± 43.6	54.9 ± 38.6	0.937
GWE (%)	96.2 ± 4.5	96.8 ± 2.6	0.483
GWIV (mmHg∙%∙mL)	10273.8 ± 2929.8	7387.1 ± 2050.2	< 0.001
GCWV (mmHg∙%∙mL)	10476.7 ± 2974.7	7496.4 ± 2044.7	< 0.001
GWWV (mmHg∙%∙mL)	215.9 ± 186.9	159.5 ± 130.3	0.158
GWEV (%∙mL)	96.9 ± 3.9	97.6 ± 2.1	0.424
**Right ventricular myocardial work**			
GWI (mmHg·%)	542.5 ± 129.0	566.6 ± 140.4	0.385
GCW (mmHg·%)	533.7 ± 127.1	560.8 ± 136.5	0.322
GWW (mmHg·%)	21.8 ± 17.3	18.7 ± 13.2	0.393
GWE (%)	93.2 ± 15.9	96.6 ± 2.4	0.289
GWIV (mmHg∙%∙mL)	3422.5 ± 1339.0	2436.6 ± 796.2	< 0.001
GCWV (mmHg∙%∙mL)	3421.5 ± 1331.3	2433.1 ± 789.6	< 0.001
GWWV (mmHg∙%∙mL)	89.6 ± 68.9	59.6 ± 41.1	0.037
GWEV (%∙mL)	94.0 ± 16.0	97.4 ± 1.6	0.294

Continuous variables are presented as means ± SD; categorical variables are reported as frequencies (%). EDVi, end-diastolic volume index; EF, ejection fraction; ESVi, end-systolic volume index; GCW, constructive myocardial work index; GCWV, volume-adjusted constructive myocardial work index; GLS, global longitudinal strain; GWE, myocardial work efficiency; GWEV, volume-adjusted myocardial work efficiency; GWI, global myocardial work index; GWIV, volume-adjusted global myocardial work index; GWW, wasted myocardial work index; GWWV, volume-adjusted wasted myocardial work index; LV, left ventricle; Mi, mass index; RV, right ventricle; SVi, stroke volume index.

Comparison between male and female athletes revealed notable 3D morphological and functional differences (Table 3). Male athletes showed higher LV EDVi, ESVi, SVi and Mi values, as well as RV EDVi, ESVi and SVi values, when compared to female athletes. Interestingly, the male sex was associated with lower resting values of LV EF, LV GLS and RV EF in comparison to the female athletes, however RV GLS did not differ when comparing the two groups. LV MW analysis results showed that while conventional MW parameters were comparable between the two groups, LV GWIV and LV GCWV were significantly greater among male athletes. Concerning the RV, similarly, only RV GWIV, RV GCWV and RV GWWV were substantially higher in male athletes compared to female athletes.

**Table 3:** 3D echocardiographic and biventricular myocardial work parameters of male and female athletes.

	Male athletes (*n* = 181)	Female athletes (*n* = 79)	*p*
Left ventricle			
LV EDVi (mL/m2)	85.5 ± 13.2	76.0 ± 10.0	< 0.001
LV ESVi (mL/m2)	37.8 ± 7.3	31.6 ± 5.6	< 0.001
LV SVi (mL/m2)	47.6 ± 7.6	44.3 ± 6.4	0.001
LV Mi (g/m2)	90.3 ± 15.6	81.1 ± 11.7	< 0.001
LV EF (%)	55.8 ± 3.9	58.2 ± 4.4	< 0.001
LV GLS (%)	-18.7 ± 2.2	-20.1 ± 1.9	< 0.001
**Right ventricle**			
RV EDVi (mL/m2)	86.4 ± 13.6	76.0 ± 11.5	< 0.001
RV ESVi (mL/m2)	39.6 ± 8.5	32.8 ± 6.7	< 0.001
RV SVi (mL/m2)	46.8 ± 7.0	43.1 ± 6.8	< 0.001
RV EF (%)	54.3 ± 4.5	56.9 ± 4.7	< 0.001
RV GLS (%)	-21.5 ± 3.4	-22.2 ± 3.5	0.139
**Left ventricular myocardial work**			
GWI (mmHg·%)	1949.3 ± 355.7	2035.1 ± 288.4	0.060
GCW (mmHg·%)	1988.5 ± 373.4	2067.8 ± 282.9	0.092
GWW (mmHg·%)	55.0 ± 44.4	57.1 ± 42.0	0.738
GWE (%)	95.9 ± 5.1	96.7 ± 2.9	0.220
GWIV (mmHg∙%∙mL)	10849.0 ± 3077.6	8955.8 ± 2029.4	< 0.001
GCWV (mmHg∙%∙mL)	11066.4 ± 3110.4	9125.6 ± 2098.0	< 0.001
GWWV (mmHg∙%∙mL)	231.4 ± 200.5	181.9 ± 148.6	0.059
GWEV (%∙mL)	96.7 ± 4.3	97.5 ± 2.5	0.138
**Right ventricular myocardial work**			
GWI (mmHg·%)	543.3 ± 130.1	540.5 ± 127.4	0.872
GCW (mmHg·%)	534.8 ± 128.1	531.3 ± 125.6	0.843
GWW (mmHg·%)	22.8 ± 18.0	19.5 ± 15.4	0.180
GWE (%)	93.4 ± 14.6	92.6 ± 18.8	0.715
GWIV (mmHg∙%∙mL)	3705.9 ± 1362.3	2762.7 ± 1019.1	< 0.001
GCWV (mmHg∙%∙mL)	3703.6 ± 1355.4	2764.4 ± 1009.4	< 0.001
GWWV (mmHg∙%∙mL)	99.7 ± 71.5	67.4 ± 57.4	< 0.001
GWEV (%∙mL)	94.3 ± 14.6	93.4 ± 18.9	0.670

Continuous variables are presented as means ± SD; categorical variables are reported as frequencies (%). EDVi, end-diastolic volume index; EF, ejection fraction; ESVi, end-systolic volume index; GCW, constructive myocardial work index; GCWV, volume-adjusted constructive myocardial work index; GLS, global longitudinal strain; GWE, myocardial work efficiency; GWEV, volume-adjusted myocardial work efficiency; GWI, global myocardial work index; GWIV, volume-adjusted global myocardial work index; GWW, wasted myocardial work index; GWWV, volume-adjusted wasted myocardial work index; LV, left ventricle; Mi, mass index; RV, right ventricle; SVi, stroke volume index.

Subsequently, we analyzed the differences between adult and adolescent athletes, adolescents displayed lower LV ESVi values than adult athletes, furthermore, LV Mi was significantly greater in adult athletes. Interestingly, LV EDVi, SVi values and RV volumes were comparable in the two age groups and did not show any substantial difference. In addition, LV and RV EF, as well as LV GLS did not show any difference between the two age groups, however, adult athletes had lower resting RV GLS values compared to adolescents (Table 4). When conducting biventricular MW analysis to compare adolescent and adult athletes, among conventional MW parameters LV GWE was lower in adolescent athletes compared to the adults. Within the novel parameters, LV GWIV, LV GCWV and LV GWEV were higher and LV GWWV were lower among adult athletes. Interestingly, age did not seem to influence the MW values of the RV, as the two age groups were comparable in terms of RV MW metrics.

**Table 4:** 3D echocardiographic and biventricular myocardial work parameters of adolescent and adult athletes.

	Adolescent athletes (*n* = 133)	Adult athletes (*n* = 127)	*p*
Left ventricle			
LV EDVi (mL/m2)	81.2 ± 13.5	84.1 ± 12.5	0.073
LV ESVi (mL/m2)	35.0 ± 7.4	36.9 ± 7.2	0.036
LV SVi (mL/m2)	46.0 ± 7.7	47.2 ± 7.2	0.214
LV Mi (g/m2)	84.5 ± 14.1	90.7 ± 15.6	< 0.001
LV EF (%)	56.9 ± 4.1	56.1 ± 4.4	0.148
LV GLS (%)	-19.4 ± 2.2	-18.9 ± 2.2	0.120
**Right ventricle**			
RV EDVi (mL/m2)	82.1 ± 14.7	84.4 ± 12.9	0.188
RV ESVi (mL/m2)	37.1 ± 8.7	38.0 ± 8.4	0.436
RV SVi (mL/m2)	44.9 ± 7.4	46.4 ± 6.7	0.104
RV EF (%)	55.0 ± 4.4	55.2 ± 5.0	0.752
RV GLS (%)	-22.2 ± 3.3	-21.2 ± 3.5	0.016
**Left ventricular myocardial work**			
GWI (mmHg·%)	1981.3 ± 352.0	1969.2 ± 325.0	0.774
GCW (mmHg·%)	1991.0 ± 376.5	2035.2 ± 319.3	0.308
GWW (mmHg·%)	61.2 ± 44.2	50.1 ± 42.5	0.050
GWE (%)	95.5 ± 5.4	96.9 ± 3.2	0.011
GWIV (mmHg∙%∙mL)	9767.0 ± 3110.3	10804.5 ± 2637.2	0.004
GCWV (mmHg∙%∙mL)	9859.4 ± 3113.9	11123.2 ± 2684.8	< 0.001
GWWV (mmHg∙%∙mL)	241.1 ± 204.8	189.9 ± 163.2	0.035
GWEV (%∙mL)	96.3 ± 4.7	97.6 ± 2.6	0.008
**Right ventricular myocardial work**			
GWI (mmHg·%)	549.2 ± 132.1	535.3 ± 125.8	0.393
GCW (mmHg·%)	540.2 ± 129.5	526.8 ± 124.6	0.404
GWW (mmHg·%)	21.6 ± 17.2	22.0 ± 17.5	0.849
GWE (%)	94.3 ± 12.3	92.0 ± 19.0	0.246
GWIV (mmHg∙%∙mL)	3343.6 ± 1382.4	3507.3 ± 1291.2	0.332
GCWV (mmHg∙%∙mL)	3339.3 ± 1375.5	3509.8 ± 1282.0	0.309
GWWV (mmHg∙%∙mL)	86.9 ± 69.5	92.4 ± 68.6	0.537
GWEV (%∙mL)	95.1 ± 12.3	92.8 ± 19.1	0.243

Continuous variables are presented as means ± SD; categorical variables are reported as frequencies (%). EDVi, end-diastolic volume index; EF, ejection fraction; ESVi, end-systolic volume index; GCW, constructive myocardial work index; GCWV, volume-adjusted constructive myocardial work index; GLS, global longitudinal strain; GWE, myocardial work efficiency; GWEV, volume-adjusted myocardial work efficiency; GWI, global myocardial work index; GWIV, volume-adjusted global myocardial work index; GWW, wasted myocardial work index; GWWV, volume-adjusted wasted myocardial work index; LV, left ventricle; Mi, mass index; RV, right ventricle; SVi, stroke volume index.

### Variations across sports disciplines

We compared the athletes by different sports classes, as shown in Supplementary Table 6. Athletes were categorized into subgroups based on their participation in mixed (*n* = 173), endurance (*n* = 63) and power (*n* = 19) sports disciplines, according to the proportion of static (isometric) and dynamic (isotonic) exercise components involved^21^.

Power athletes had the longer competition history, averaging 19 years, while endurance athletes reported significantly higher weekly training volumes with an average of 22 hours, and further demonstrated the highest peak exercise capacity among all groups. When comparing 3DE metrics, LV Mi was notably lower in power athletes compared to the other two sports disciplines, however LV EDVi, ESVi and SVi were similar across study groups. Endurance athletes showed the highest RV ESVi values, while RV EDVi and SVi did not differ among the groups. Concerning the LV function, the sports groups were interestingly comparable, on the contrary, regarding the RV function, endurance athletes exhibited the lower resting RV EF and GLS.

After performing MW analysis, as for the LV, endurance athletes showed the lower conventional LV GWW and the higher LV volume-adjusted GWE values. Interestingly, other conventional and volume-adjusted LV MW parameters, such as LV GWI and LV GCW or LV GWIV and LV GCWV were comparable between groups. However, concerning the RV, among conventional RV MW metrics, endurance athletes exhibited lower RV GWE values. When analyzing the volume-adjusted RV MW parameters, power athletes showed lower values of RV GWIV and RV GCWV, while the mixed class portrayed the higher values of these parameters. Furthermore, endurance athletes presented lower RV GWEV values.

### Correlation of resting echocardiographic measures and myocardial work parameters with peak exercise capacity

Univariable correlations were assessed between demographic variables along with 3DE-derived parameters and VO_2_/kg in the study’s athlete population (Supplementary Table 7). Being an endurance athlete correlated with higher peak exercise capacity. Concerning the left heart, LV volumes, such as LV EDVi, LV ESVi and LV SVi, furthermore, LV Mi correlated significantly with peak exercise capacity. The functional parameters of the LV, such as LV EF and LV GLS showed a weaker and inverse correlation with VO_2_/kg. As for MW parameters, conventional LV GWI and GCW portrayed a weak inverse correlation, and among the volume-adjusted parameters, LV GWIV and LV GCWV also showed a weak but direct correlation with VO_2_/kg.

Regarding the RV, similarly, RV volumes, such as RV EDVi, RV ESVi and RV SVi exhibited notable correlation with VO_2_/kg. While RV GLS did not show any correlation, RV EF showed a weak inverse correlation with peak exercise capacity. Concerning the RV, conventional RV GWI, RV GCW and RV GWW exhibited a weak correlation, while interestingly, the novel RV GWIV, RV GCWV and RV GWWV parameters showed a stronger direct correlation with VO_2_/kg.

To identify independent predictors of VO₂/kg in the athlete group, we conducted multiple linear regression analysis in the athlete cohort (Table 5). The model included basic demographic variables (age, sex, BSA), HR, endurance sports class, and 3D LV and RV morphological and functional parameters, specifically LV EDVi, LV EF, RV EDVi, and RV EF, as well as LV GWIV and RV GWIV. Our findings revealed that besides sex, BSA, HR and endurance sports class, only RV GWIV emerged as an independent predictor of exercise capacity with a cumulative R value of 0.684 (< 0.001), while age, LV EDVi, LV EF, LV GWIV, RV EDVi, RV EF were not independent predictors.

**Table 5:** Multivariate linear regression analysis: independent predictors of VO_2_/kg.

Covariates	β	*P*
Age	-0.075	0.189
Sex	-0.495	< 0.001
BSA	-0.295	< 0.001
HR	-0.118	0.032
Endurance sports class	0.306	< 0.001
LV EDVi	0.168	0.118
LV EF	-0.073	0.282
LV GWIV	-0.011	0.894
RV EDVi	-0.043	0.698
RV EF	-0.068	0.389
RV GWIV	0.186	0.012
**Cumulative R**	0.684	
**Standard error**	5.72%	
**Cumulative** ***P***	< 0.001	

EDVi, end-diastolic volume index; EF, ejection fraction; GWIV, volume-adjusted global myocardial work index; LV, left ventricle; RV, right ventricle.

## Discussion

**Fig. 2:**
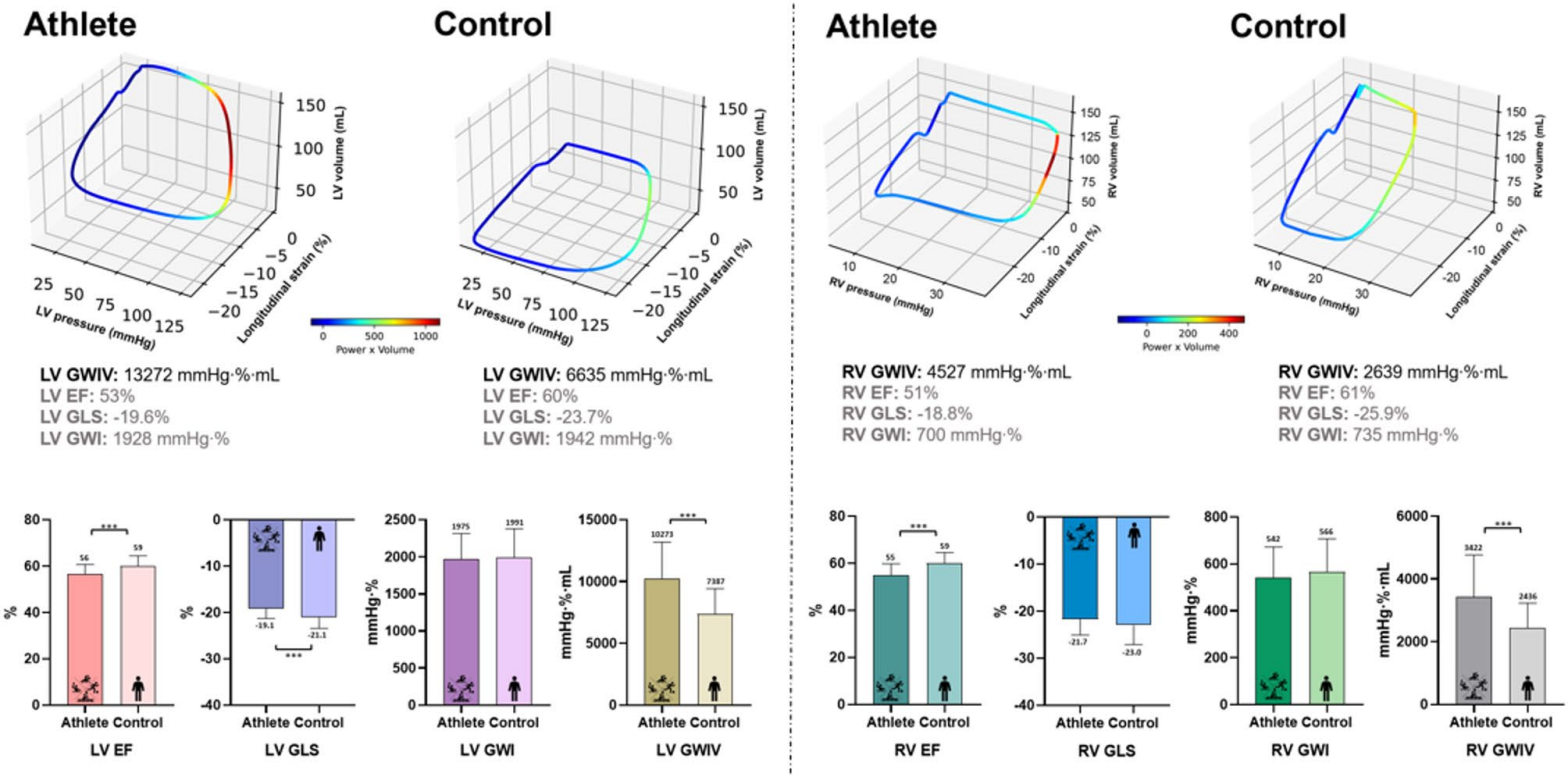
Comparison of biventricular systolic functional parameters between competitive athletes and sedentary healthy controls. Top panels show representative 3D pressure-strain-volume loops for the left and right ventricles in an athlete and in a control subject. Bottom panels display group comparisons for left and right ventricular systolic functional parameters. EF, ejection fraction; GLS, global longitudinal strain; GWI, global myocardial work index; GWIV, volume-adjusted global myocardial work index; LV, left ventricle; RV, right ventricle.

To the best of our knowledge, this study represents the first comprehensive evaluation of noninvasive, 3D biventricular MW indices in a large cohort of competitive athletes, with a particular focus on RV MW, which remains notably under-explored in current literature. Unlike conventional echocardiographic approaches, our proposed metrics of volume-adjusted global MW provided refined insight into enhanced cardiac performance in competitive athletes, even during resting conditions. While conventional metrics of systolic function, such as 3D EF and GLS, showed lower values in athletes, and PS loop-derived MW analysis revealed no significant differences between athletes and controls, our novel, PSV loop-derived biventricular volume-adjusted MW parameters were significantly higher among athletes (Fig. 2). Among systolic functional metrics, RV GWIV showed the strongest correlation with exercise capacity. Furthermore, in contrast to other echocardiographic metrics, RV GWIV was an independent predictor of peak VO₂/kg in multivariate logistic regression analysis.

Adaptive cardiac remodeling in response to long-term intensive training results in distinct structural and functional alterations of the athlete’s heart, primarily characterized by increased chamber volumes, enhanced stroke volume, and optimized mechanical efficiency, all of which support the elevated cardiac output demands during exercise, typically observed in endurance athletes^22,23^. However, characterizing functional adaptation in this context remains challenging due to the substantially altered hemodynamic profiles in athletes. Furthermore, despite the observed favorable physiological changes, conventional resting systolic indices such as EF and GLS often fall within the lower normal range in highly trained individuals, which may be misinterpreted as myocardial dysfunction even in the absence of overt disease. This may be partially attributable to the inherent load-dependency of these conventional markers of systolic function, reflecting ventriculo-arterial coupling rather than myocardial contractile performance^6,11,13,22,24^. This was further recognized by recent studies using advanced 2D and 3D speckle-tracking echocardiography, revealing that the augmented myocardial function of the athlete’s heart often remains masked when using load-sensitive parameters^25^. Endurance training, in particular, is associated with a marked increase in both cardiac output and systolic blood pressure, resulting in a combined LV volume- and pressure overload during peak performance. Notably, the same hemodynamic milieu applies to the RV, as the right heart is also exposed to the same cardiac output demands, and evidence suggests that pulmonary pressures also increase substantially during exercise. In order to enable this often-exceptional hemodynamic stress, significant adaptive changes of cardiac geometry and function occur. Still, compared to other hemodynamic overload states, the cellular pathways of this adaptive process are quite unique, and even more importantly, the hemodynamic derangements that drive this extensive remodeling are not persistently present. Therefore, the athlete’s heart at rest may be perceived as a severely underfilled, on the other hand, markedly remodeled heart, leading to changes in conventional systolic metrics that may appear reduced despite preserved or even enhanced myocardial contractility, instead of reflecting true contractile impairment, a phenomenon most commonly observed in endurance sports^22^. Ilardi et al. noted that, as EF and GLS often fail to capture subtle changes of contractility, relying solely on these parameters could be misleading, as they partly reflect the coupling between the heart and the vascular system in the context of the distinct loading conditions characteristic of athletic physiology^24,26^. Consistent with these concepts, our findings also showed that athletes had lower resting values of EF and GLS compared to controls, whereas the volume-adjusted MW metrics were consistently higher^24,26^. These results suggest that load-sensitive parameters may underrepresent myocardial contractile efficiency in trained individuals, and that incorporating volume into the pressure-strain relationship may offer a physiologically more comprehensive approach.

Invasive PV loop analysis remains the gold standard for assessing intrinsic myocardial contractility. Previous research in humans and animal models consistently demonstrated that PV analysis provides quantitative metrics that reflect the heart’s intrinsic inotropy incomparable to traditional echocardiographic markers^27–29^. Specifically, while PV loop assessment has been investigated in patients with pulmonary hypertension (PH), the underlying hemodynamic characteristics in PH differ substantially from those seen in athletes. Moreover, routine application of invasive PV analysis in athletic populations is not feasible due to its procedural burden^30^. Although preliminary studies have explored noninvasive single-beat PV estimation, its applicability and physiological relevance in the context of the athlete’s heart remain limited. Östenson et al. demonstrated that LV PV loops can be generated noninvasively using cardiac magnetic resonance imaging combined with brachial blood pressure measurements during exercise in both endurance-trained athletes and sedentary controls. Their findings showed that exercise is associated with enhanced ventricular efficiency and contractility, accompanied by reductions in afterload and ventriculo-arterial coupling. These results support known physiological responses and highlight the potential of this approach for assessing cardiac hemodynamics in athletes and other populations^31^. Conversely, recently introduced, noninvasive MW analysis, especially for LV functional assessment, has emerged as a promising, widely used, and validated method of assessing cardiac performance. Applying to the LV, Russell and colleagues’ method utilizes normalized reference pressure curves adjusted to individual valve timing intervals and scaled according to measured blood pressure^13^. Our methodology builds upon this foundation, extending this concept to the RV using an AI-based integrative tool^18^. Even to this day only a handful of prior publication utilizes RV MW analysis, however in the LV more recently experimental studies reported that LV GWI and GCW correlate more strongly with gold standard contractility measures than GLS alone, emphasizing the added value of MW in capturing myocardial function^32,33^. While pressure-driven changes dominate in pathological conditions such as PH, athletic remodeling is primarily volume-driven, underscoring the distinct hemodynamic profile of this population. Importantly, from a mechanistic standpoint, applying single-beat pressure-volume loop estimation may not fully capture the physiological adaptations seen in endurance athletes. As commonly seen in this population, chamber volume increases while pressure remains normal or slightly elevated, and as a result, the end-systolic pressure-volume relationship may become less steep, potentially suggesting reduced contractility, even in the presence of preserved or enhanced myocardial performance. Consequently, Ees may underrepresent contractile function in athletes, reflecting similar limitations as EF. Indeed, animal studies have demonstrated improved intrinsic contractility despite structural remodeling^33^, findings not typically captured by resting single-beat PV analyses in humans.

Our novel parameter integrates information from three different domains: myocardial deformation, systemic load, and pressure relations. It is also important to emphasize that strain not only reflects longitudinal shortening but also provides insight into myocardial mechanics. This integrated pressure-strain-volume framework enables a more physiologically comprehensive assessment of systolic function, particularly under the altered loading conditions, as our proposed measure hypothesizes independence from both afterload (through strain-based MW) and preload/volume load (through the multiplicative incorporation of instantaneous volume).

In our study, both LV and RV volume-adjusted MW metrics were significantly higher in athletes compared to controls, while interestingly, conventional PS loop-derived MW parameters did not differ between the groups. This finding suggests that adjusting for preload, especially in the context of physiological chamber dilation, the PSV equation may help uncover the athletes’ contractile state more accurately. Of note, our findings align with previous results by Tokodi et al., demonstrating a strong correlation between noninvasively derived MW indices adjusted to LV cavity size and invasively acquired PV loop-derived measures, in a rodent model of volume overload-induced LV dysfunction^12^.

Our subgroup analyses revealed additional insights into the heterogeneity of functional remodeling among athletes, influenced by demographic and training-specific characteristics. Male athletes exhibited larger biventricular volumes, lower values of EF and GLS, and higher values of biventricular volume-adjusted MW metrics, aligning with known sex-based differences in exercise-induced cardiovascular remodeling^5,34,35^. Regarding age-related differences, adult athletes displayed higher values of LV GWIV and GCWV compared to adolescents, potentially reflecting more advanced structural remodeling and myocardial adaptation from prolonged training exposure^36^. In contrast, in the RV, both conventional and volume-adjusted MW indices were mostly unaffected by age, indicating that RV functional adaptation may occur earlier or is less influenced by cumulative training load^5,35,37^. Once an initial adaptation occurs (often in adolescence), additional years of intensive training may contribute less to resting RV function. Overall, these findings imply that older athletes exhibit greater functional capacity overall, while the functional RV adaptation process appears to plateau earlier, highlighting a temporal asymmetry in cardiac plasticity^38^.

In our study, the athlete cohort included individuals from endurance, power, and mixed sports classes, each imposing slightly different hemodynamic and mechanical stimuli. This diversity generates a wide spectrum of cardiac remodeling and provides a unique framework to explore how varying training types and intensities influence structural and functional cardiac adaptations. When comparing sports disciplines, expected distinctions emerged. Endurance-trained athletes achieved the highest peak oxygen uptake and demonstrated lower resting RV EF and strain values, consistent with the chronic volume load associated with intensive aerobic exercise. This resonates with the findings also noted by Oxborough et al., reporting that extreme endurance training can lead to borderline decreased RV systolic function at rest^25^. Interestingly, the observed differences in LV MW assessment among sports types were negligible, whereas in the RV, MW analysis revealed characteristic differences aligning with prior findings, describing that extreme aerobic exercise could put strain on the RV and may cause subtle inefficiencies or delayed relaxation^13^. Overall, these sports-specific findings emphasize that both the magnitude and nature of cardiac hemodynamic stress (volume vs. pressure) drive distinct remodeling patterns, underscoring the value of MW analysis in detecting even subtle effects on myocardial mechanics^36,39^. Therefore, incorporating such nuanced evaluation could help refine sports cardiology screening by providing benchmarks tailored to an athlete’s discipline.

Recent investigations have increasingly focused on LV MW in athlete populations, evaluating its relationship with training adaptations and its additive value in the assessment of systolic function^6,40,41^. A recent publication by Tokodi et al. provided an in-depth translational outlook on MW in athletes; in a rodent model of physiological hypertrophy induced by swim training, invasive PV analysis demonstrated that noninvasive MW indices reliably reflected intrinsic contractility compared to GLS. It is important to emphasize that in these rodent models, exercise-induced cardiac remodeling is predominantly characterized by concentric hypertrophy without significant changes in cavity size, implying that chamber geometry, and consequently preload, have a limited impact on systolic function. In a parallel human study involving elite swimmers, despite decreased values of GLS and elevated blood pressure, LV GWI was increased at rest and showed a strong correlation with peak oxygen uptake (VO₂max)^6^, latter result consistent with our findings.

A particularly novel insight of our study is the pivotal role of RV contractile performance in determining aerobic exercise capacity. While LV function has traditionally been the primary focus in exercise physiology, the RV’s capacity to support pulmonary circulation becomes critically important during physical exertion yet remains under-investigated in athlete populations^22,42^. Under healthy resting conditions, the RV operates under low pressure and high efficiency, accommodating venous return with minimal effort. During intense exercise, however, the RV must dramatically augment its stroke work to sustain forward flow through the pulmonary circulation with increasing pulmonary vascular resistance. Our findings show that RV volume-adjusted GWI had a strong correlation with peak VO₂/kg and was an independent echocardiographic predictor of exercise capacity in multivariate analysis. This aligns with emerging evidence across both health and disease; in patients with heart failure or PH, invasive assessments have shown that RV contractility indices and RV-pulmonary arterial coupling metrics correlate strongly with exercise capacity and prognosis^22^. In parallel, athlete-specific studies have reported that a healthy RV can enhance its contractile performance during peak exercise, reaching pulmonary pressures that would be considered pathologically elevated at rest, without evidence of functional decompensation^22,43,44^. Additionally, previous data from invasive hemodynamic studies in both athletes and PH patients have demonstrated that invasive RV hemodynamic measures, such as Ees and RV-arterial coupling (Ees/Ea), are closely associated with peak exercise capacity, even in distinct pathophysiological settings^6,45–47^. Similarly, our findings demonstrated that RV GWIV and GCWV strongly correlated with exercise capacity, whereas their LV counterparts exhibited only weak correlations. Notably, RV GWIV remained an independent predictor of peak exercise capacity in multivariate analysis, emphasizing its potential as a noninvasive marker of physiologic reserve in athletic screening. In practical terms, an RV with greater work capacity could more effectively sustain enhanced pulmonary perfusion and facilitate oxygen uptake during exercise, which represents an essential determinant of aerobic capacity, a concept supported by La Gerche et al., who demonstrated exercise-induced changes in RV function among endurance athletes and emphasized that the interaction between the RV and the pulmonary circulation may represent a critical factor of exercise tolerance^43,47^. These findings underscore the clinical value of comprehensive, advanced echocardiographic evaluation of RV function alongside traditional LV-focused assessments.

### Clinical implications and future directions

Our findings indicate that incorporating PSV-loop-derived MW analysis into routine echocardiographic evaluation can greatly enhance the functional assessment of athletes. Specifically, GWIV and GCWV may serve as valuable tools to differentiate physiological from pathological adaptations in individuals with borderline functional indices or structural remodeling falling within the “grey zone.” Moreover, beyond athletic populations, these novel parameters may also prove useful in broader clinical settings, offering a means to detect subtle deviations from normal physiological adaptation and potentially identify early signs of cardiomyopathy. While promising, our findings also highlight the need for longitudinal studies to explore how GWIV and related parameters evolve over time, particularly in relation to training load, detraining, and pathological remodeling. Additionally, further validation against invasive gold-standard measures of contractility in human subjects will be crucial to confirm the clinical applicability and reliability of these noninvasive metrics.

### Limitations

Several limitations should be acknowledged. First, the retrospective nature of the study and the selection bias due to a competitive athlete cohort limit wider applicability. Second, although the sample sizes were imbalanced, the large athlete cohort and use of advanced 3D imaging still provide valuable insights despite this limitation. Third, although the novel PSV-loop generation was enabled by proprietary software, it may not yet be widely accessible or standardized, potentially limiting its clinical interpretation and use. While our approach of noninvasive estimation of right-sided pressures is based on a previously published and validated methodology^18^, its broader applicability and generalizability may be limited, highlighting the need for continued refinement and validation of noninvasive alternatives. Efforts to standardize PSV-loop-based analysis, possibly through open-source platforms or multicenter validation initiatives, will be critical for broader use.

## Conclusion

By analyzing the left and right ventricular pressure-strain-volume relationship, volume-adjusted myocardial work indices may provide a more accurate reflection of the enhanced biventricular systolic function in athletes, even during resting conditions. Moreover, volume-adjusted right ventricular global myocardial work was independently associated with aerobic capacity, pointing towards the RV’s importance in exercise-induced functional remodeling. In the long term, these findings may contribute to the refinement of echocardiographic assessment in competitive athletes and support more precise monitoring in sports cardiology. Additionally, it may assist in cases falling within the diagnostic “grey zone,” where it is not evident whether training-induced adaptations are purely physiological or indicative of emerging pathological changes.

## Supplementary Information

Below is the link to the electronic supplementary material.

Supplementary Material 1

## Data Availability

The data underlying this article will be made available by the corresponding author (Andrea Ferencz M.D., ferencz.andrea12@gmail.com) upon reasonable request.

## Abbreviations

2DE: two–dimensional echocardiography
3DE: three–dimensional echocardiography
A: mitral inflow velocity during atrial contraction
a’: peak late (atrial) diastolic annular velocity
BSA: body surface area
CPET: cardiopulmonary exercise testing
DT: deceleration time
E: early diastolic mitral inflow velocity
e’: early diastolic annular velocity
Ea: arterial elastance
ECG: electrocardiogram
EDVi: end–diastolic volume indexed to body surface area
Ees: end–systolic elastance
EF: ejection fraction
ESVi: end–systolic volume indexed to body surface area
FAC: fractional area change
GCW: constructive myocardial work index
GCWV: volume–adjusted constructive myocardial work index
GLS: global longitudinal strain
GWE: myocardial work efficiency
GWEV: volume–adjusted myocardial work efficiency
GWI: global myocardial work index
GWIV: volume–adjusted global myocardial work index
GWW: wasted myocardial work index
GWWV: volume–adjusted wasted myocardial work index
HR: heart rate
IVSd: interventricular septal thickness at end–diastole
LAVi: left atrial volume index
LV: left ventricle
LVIDd: LV internal diameter at end–diastole
LVIDs: LV internal diameter at end–systole
Mi: mass index
MW: myocardial work
PASP: pulmonary artery systolic pressure
PH: pulmonary hypertension
PRSW: preload recruitable stroke work
PS: pressure–strain
PSV: pressure–strain–volume
PV: pressure–volume
PWd: posterior wall thickness at end–diastole
RAVi: right atrial volume index
RV: right ventricle
RVd: RV basal diameter
RVFWLS: RV free wall longitudinal strain
RVSLS: RV septal longitudinal strain
RWT: relative wall thickness
s’: systolic annular velocity
SBP: systolic blood pressure
STE: speckle–tracking echocardiography
SVi: stroke volume indexed to body surface area
TAPSE: tricuspid annular plane systolic excursion
VO_2_: peak oxygen uptake
VO_2_/kg: peak oxygen uptake indexed to body weight
