# Supplementary material for: Noninvasive biventricular pressure-strain-volume loop-derived myocardial work analysis in competitive athletes
Source: Sci Rep. 2026 Jan 8;16:4848. doi: 10.1038/s41598-026-35206-0 (PMC12873281; doi:10.1038/s41598-026-35206-0)
Supplement: Supplementary file 1 — Supplementary Material 1 [file 41598_2026_35206_MOESM1_ESM.docx]

**Supplemental Data**

**Supplementary Table 1**. Baseline and training-specific characteristics of male and female athletes

|  | **Male athletes (*n* = 181)** | **Female athletes (*n* = 79)** | **p** |
| --- | --- | --- | --- |
| **Baseline characteristics** | | | |
| Age (years) | 19.8 ± 6.0 | 20.6 ± 4.9 | 0.262 |
| Competitive training since (years) | 12.5 ± 5.9 | 11.0 ± 4.9 | 0.048 |
| Training time (h/week) | 13.9 ± 6.5 | 19.7 ± 7.5 | <0.001 |
| VO_2_ (L/min) | 4.5 ± 0.8 | 3.1 ± 0.4 | <0.001 |
| VO_2_/kg (mL/kg/min) | 56.0 ± 7.1 | 47.7 ± 5.3 | <0.001 |
| **Type of sport** | | | |
| Mixed, n (%) | 128 (70.7) | 45 (56.9) | - |
| Endurance, n (%) | 38 (20.9) | 25 (31.6) | - |
| Power, n (%) | 12 (6.6) | 7 (8.8) | - |
| Skill, n (%) | 3 (1.6) | 2 (2.5) | - |

Continuous variables are presented as means ± SD; categorical variables are reported as frequencies (%). VO2, peak oxygen uptake; VO2/kg, peak oxygen uptake indexed to body weight.

**Supplementary Table 2**. Baseline and training-specific characteristics of adolescent and adult athletes

|  | **Adolescent athletes (*n* = 133)** | **Adult athletes (*n* = 127)** | **p** |
| --- | --- | --- | --- |
| **Baseline characteristics** | | | |
| Age (years) | 15.7 ± 1.5 | 24.5 ± 5.1 | <0.001 |
| Male, *n* (%) | 95 (71.4) | 86 (67.7) | 0.606 |
| Competitive training since (years) | 8.6 ± 3.0 | 15.6 ± 5.5 | <0.001 |
| Training time (h/week) | 12.8 ± 6.5 | 18.6 ± 7.0 | <0.001 |
| VO_2_ (L/min) | 3.6 ± 0.8 | 4.0 ± 0.9 | 0.001 |
| VO_2_/kg (mL/kg/min) | 54.8 ± 6.9 | 52.1 ± 8.1 | 0.004 |
| **Type of sport** | | | |
| Mixed, n (%) | 108 (81.2) | 65 (51.1) | - |
| Endurance, n (%) | 19 (14.2) | 44 (34.6) | - |
| Power, n (%) | 3 (2.2) | 16 (12.5) | - |
| Skill, n (%) | 3 (2.2) | 2 (1.5) | - |

Continuous variables are presented as means ± SD; categorical variables are reported as frequencies (%). VO2, peak oxygen uptake; VO2/kg, peak oxygen uptake indexed to body weight.

**Supplementary Table 3.** Conventional 2D echocardiographic parameters of athlete and control groups

|  | **Athletes (*n* = 260)** | **Controls (*n* = 24)** | **p** |
| --- | --- | --- | --- |
| **Left ventricle** | | | |
| LVIDd (mm) | 50.6 ± 4.8 | 45.4 ± 4.2 | <0.001 |
| LVIDs (mm) | 34.7 ± 5.1 | 27.8 ± 1.9 | 0.003 |
| IVSd (mm) | 9.8 ± 1.7 | 8.3 ± 1.3 | <0.001 |
| PWd (mm) | 8.7 ± 1.3 | 7.2 ± 0.8 | <0.001 |
| RWT (%) | 0.3 ± 0.05 | 0.3 ± 0.03 | 0.018 |
| LV Mi (g/m^2^) | 90.2 ± 19.7 | 66.2 ± 14.6 | <0.001 |
| E (cm/s) | 86.3 ± 18.1 | 98.7 ± 15.7 | 0.001 |
| A (cm/s) | 53.8 ± 13.5 | 64.0 ± 17.1 | <0.001 |
| E/A ratio | 1.6 ± 0.44 | 1.6 ± 0.45 | 0.673 |
| DT (ms) | 173.7 ± 37.0 | 154.2 ± 26.6 | 0.012 |
| Mitral lateral s′ (cm/s) | 11.8 ± 2.3 | 12.6 ± 2.6 | 0.115 |
| Mitral lateral e′ (cm/s) | 18.0 ± 3.3 | 20.5 ± 2.9 | <0.001 |
| Mitral lateral a′ (cm/s) | 6.7 ± 1.9 | 7.5 ± 2.4 | 0.047 |
| Mitral medial s′ (cm/s) | 9.1 ± 1.4 | 9.7 ± 1.7 | 0.051 |
| Mitral medial e′ (cm/s) | 13.3 ± 2.4 | 14.6 ± 2.2 | 0.010 |
| Mitral medial a′ (cm/s) | 7.0 ± 1.7 | 7.3 ± 2.1 | 0.351 |
| E/e′ average | 5.7 ± 1.1 | 5.8 ± 0.8 | 0.634 |
| LAVi (ml/m^2^) | 27.2 ± 7.7 | 21.8 ± 5.7 | 0.001 |
| **Right ventricle** | | | |
| RVd (mm) | 33.8 ± 4.1 | 30.7 ± 3.4 | <0.001 |
| PASP (mmHg) | 24.1 ± 4.0 | 23.5 ± 3.9 | 0.488 |
| TAPSE (mm) | 24.7 ± 4.7 | 23.8 ± 6.7 | 0.383 |
| FAC (%) | 47.7 ± 6.1 | 52.4 ± 6.8 | <0.001 |
| RVSLS (%) | -22.5 ± 4.8 | -22.5 ± 5.9 | 0.988 |
| RVFWLS (%) | -29.4 ± 4.0 | -31.6 ± 4.7 | 0.011 |
| RAVi (ml/m^2^) | 28.4 ± 7.2 | 25.1 ± 8.3 | 0.032 |

Continuous variables are presented as means ± SD, categorical variables are reported as frequencies (%). A: mitral inflow velocity during atrial contraction, a’: peak late (atrial) diastolic annular velocity, DT: deceleration time, E: early diastolic mitral inflow velocity, e’: early diastolic annular velocity, FAC: fractional area change, IVSd: interventricular septal thickness at end-diastole, LAVi: left atrial volume index, LV: left ventricle, LVIDd: LV internal diameter at end-diastole, LVIDs: LV internal diameter at end-systole, Mi: mass index, PASP: pulmonary artery systolic pressure, PWd: posterior wall thickness at end-diastole, RAVi: right atrial volume index, RV: right ventricle, RVd: RV basal diameter, RVFWLS: RV free wall longitudinal strain, RVSLS: RV septal longitudinal strain, RWT: relative wall thickness, s’: systolic annular velocity, TAPSE: tricuspid annular plane systolic excursion.

**Supplementary Table 4.** Conventional 2D echocardiographic parameters of male and female athletes

|  | **Male athletes (*n* = 181)** | **Female athletes (*n* = 79)** | **p** |
| --- | --- | --- | --- |
|  |  |  |  |
| **Left ventricle** | | | |
| LVIDd (mm) | 51.4 ± 5.2 | 48.6 ± 3.2 | <0.001 |
| LVIDs (mm) | 35.9 ± 5.4 | 33.1 ± 4.0 | 0.021 |
| IVSd (mm) | 10.2 ± 1.7 | 8.9 ± 1.2 | <0.001 |
| PWd (mm) | 9.0 ± 1.3 | 8.0 ± 1.0 | <0.001 |
| RWT (%) | 0.3 ± 0.05 | 0.3 ± 0.04 | <0.001 |
| LV Mi (g/m^2^) | 94.8 ± 20.2 | 79.5 ± 13.5 | <0.001 |
| E (cm/s) | 86.0 ± 17.8 | 87.0 ± 18.8 | 0.700 |
| A (cm/s) | 53.2 ± 12.3 | 55.1 ± 16.0 | 0.300 |
| E/A ratio | 1.6 ± 0.45 | 1.6 ± 0.43 | 0.610 |
| DT (ms) | 170.6 ± 34.3 | 180.5 ± 41.9 | 0.047 |
| Mitral lateral s′ (cm/s) | 11.9 ± 2.4 | 11.4 ± 2.1 | 0.156 |
| Mitral lateral e′ (cm/s) | 17.6 ± 3.3 | 18.9 ± 3.2 | 0.003 |
| Mitral lateral a′ (cm/s) | 6.5 ± 1.9 | 7.1 ± 1.8 | 0.024 |
| Mitral medial s′ (cm/s) | 8.9 ± 1.3 | 9.5 ± 1.4 | 0.002 |
| Mitral medial e′ (cm/s) | 12.8 ± 2.3 | 14.4 ± 2.4 | <0.001 |
| Mitral medial a′ (cm/s) | 6.8 ± 1.6 | 7.3 ± 1.8 | 0.036 |
| E/e′ average | 5.8 ± 1.08 | 5.3 ± 1.22 | <0.001 |
| LAVi (ml/m^2^) | 26.6 ± 7.7 | 28.5 ± 7.8 | 0.062 |
| **Right ventricle** | | | |
| RVd (mm) | 34.6 ± 3.8 | 32.0 ± 4.0 | <0.001 |
| PASP (mmHg) | 24.5 ± 4.0 | 23.1 ± 3.9 | 0.015 |
| TAPSE (mm) | 24.9 ± 4.7 | 24.3 ± 4.6 | 0.338 |
| FAC (%) | 47.0 ± 5.9 | 49.3 ± 6.2 | 0.005 |
| RVSLS (%) | -22.0 ± 4.7 | -23.6 ± 4.7 | 0.017 |
| RVFWLS (%) | -29.0 ± 3.9 | -30.3 ± 4.1 | 0.020 |
| RAVi (ml/m^2^) | 29.1 ± 7.1 | 26.9 ± 7.2 | 0.021 |

Continuous variables are presented as means ± SD, categorical variables are reported as frequencies (%). A: mitral inflow velocity during atrial contraction, a’: peak late (atrial) diastolic annular velocity, DT: deceleration time, E: early diastolic mitral inflow velocity, e’: early diastolic annular velocity, FAC: fractional area change, IVSd: interventricular septal thickness at end-diastole, LAVi: left atrial volume index, LV: left ventricle, LVIDd: LV internal diameter at end-diastole, LVIDs: LV internal diameter at end-systole, Mi: mass index, PASP: pulmonary artery systolic pressure, PWd: posterior wall thickness at end-diastole, RAVi: right atrial volume index, RV: right ventricle, RVd: RV basal diameter, RVFWLS: RV free wall longitudinal strain, RVSLS: RV septal longitudinal strain, RWT: relative wall thickness, s’: systolic annular velocity, TAPSE: tricuspid annular plane systolic excursion.

**Supplementary Table 5.** Conventional 2D echocardiographic parameters of adolescent and adult athletes

|  | **Adolescent athletes (*n* = 133)** | **Adult athletes (*n* = 127)** | **p** |
| --- | --- | --- | --- |
| **Left ventricle** | | | |
| LVIDd (mm) | 49.4 ± 4.3 | 51.8 ± 5.0 | <0.001 |
| LVIDs (mm) | 32.2 ± 3.7 | 35.6 ± 5.2 | 0.013 |
| IVSd (mm) | 9.4 ± 1.4 | 10.2 ± 1.8 | <0.001 |
| PWd (mm) | 8.4 ± 1.2 | 8.9 ± 1.3 | <0.001 |
| RWT (%) | 0.3 ± 0.05 | 0.3 ± 0.05 | 0.359 |
| LV Mi (g/m^2^) | 86.2 ± 16.5 | 94.3 ± 21.8 | <0.001 |
| E (cm/s) | 91.0 ± 16.9 | 81.6 ± 18.1 | <0.001 |
| A (cm/s) | 56.3 ± 14.3 | 51.3 ± 12.2 | 0.002 |
| E/A ratio | 1.6 ± 0.4 | 1.6 ± 0.4 | 0.407 |
| DT (ms) | 170.3 ± 36.6 | 177.1 ± 37.2 | 0.142 |
| Mitral lateral s′ (cm/s) | 11.8 ± 2.1 | 11.8 ± 2.5 | 0.985 |
| Mitral lateral e′ (cm/s) | 18.2 ± 3.2 | 17.9 ± 3.4 | 0.461 |
| Mitral lateral a′ (cm/s) | 6.2 ± 1.8 | 7.3 ± 1.7 | <0.001 |
| Mitral medial s′ (cm/s) | 9.0 ± 1.4 | 9.2 ± 1.4 | 0.233 |
| Mitral medial e′ (cm/s) | 13.5 ± 2.2 | 13.0 ± 2.6 | 0.109 |
| Mitral medial a′ (cm/s) | 6.7 ± 1.6 | 7.3 ± 1.7 | 0.011 |
| E/e′ average | 5.9 ± 1.1 | 5.4 ± 1.5 | 0.002 |
| LAVi (ml/m^2^) | 25.4 ± 7.9 | 29.0 ± 7.1 | <0.001 |
| **Right ventricle** | | | |
| RVd (mm) | 33.5 ± 4.0 | 34.1 ± 4.2 | 0.206 |
| PASP (mmHg) | 23.8 ± 4.0 | 24.3 ± 4.1 | 0.399 |
| TAPSE (mm) | 24.6 ± 4.5 | 24.8 ± 4.9 | 0.700 |
| FAC (%) | 47.1 ± 6.1 | 48.3 ± 6.1 | 0.124 |
| RVSLS (%) | -23.2 ± 4.6 | -21.8 ± 4.9 | 0.019 |
| RVFWLS (%) | -29.5 ± 3.9 | -29.3 ± 4.2 | 0.580 |
| RAVi (ml/m^2^) | 26.6 ± 6.8 | 30.3 ± 7.1 | <0.001 |

Continuous variables are presented as means ± SD, categorical variables are reported as frequencies (%). A: mitral inflow velocity during atrial contraction, a’: peak late (atrial) diastolic annular velocity, DT: deceleration time, E: early diastolic mitral inflow velocity, e’: early diastolic annular velocity, FAC: fractional area change, IVSd: interventricular septal thickness at end-diastole, LAVi: left atrial volume index, LV: left ventricle, LVIDd: LV internal diameter at end-diastole, LVIDs: LV internal diameter at end-systole, Mi: mass index, PASP: pulmonary artery systolic pressure, PWd: posterior wall thickness at end-diastole, RAVi: right atrial volume index, RV: right ventricle, RVd: RV basal diameter, RVFWLS: RV free wall longitudinal strain, RVSLS: RV septal longitudinal strain, RWT: relative wall thickness, s’: systolic annular velocity, TAPSE: tricuspid annular plane systolic excursion.

|  | **Mixed (*n* = 173)** | **Endurance (*n* = 63)** | **Power (*n* = 19)** | **ANOVA P** |
| --- | --- | --- | --- | --- |
| Age (years) | 20.1 ± 6.8**^#§^** | 23.6 ± 4.7***^*^*^§^** | 27.7 ± 5.8***^*^*^#^** | <0.001 |
| Male, n (%) | 128 (73.9) | 38 (60.3) | 12 (63.1) | 0.104 |
| Competitive training since (years) | 11.4 ± 7.1**^#§^** | 16.9 ± 4.8***^*^*** | 18.7 ± 6.4***^*^*** | <0.001 |
| Training time (h/week) | 14.8 ± 7.8**^#^** | 21.6 ± 5.6***^*^*^§^** | 15.5 ± 4.4**^#^** | <0.001 |
| VO_2_/kg (mL/kg/min) | 48.8 ± 6.5**^#§^** | 58.8 ± 5.9***^*^*^§^** | 45.6 ± 3.4***^*^*^#^** | <0.001 |
| **Left Ventricle** | | | | |
| LV EDVi (mL/m^2^) | 83.2 ± 12.6 | 82.3 ± 13.7 | 79.5 ± 12.1 | 0.516 |
| LV ESVi (mL/m^2^) | 36.1 ± 7.0 | 36.1 ± 8.4 | 35.2 ± 6.0 | 0.882 |
| LV SVi (mL/m^2^) | 46.9 ± 7.5 | 46.2 ± 7.0 | 44.3 ± 6.9 | 0.357 |
| LV Mi (g/m^2^) | 88.1 ± 14.6**^§^** | 87.8 ± 14.9**^§^** | 79.0 ± 10.9***^*^*^#^** | 0.049 |
| LV EF (%) | 56.5 ± 4.2 | 56.4 ± 4.9 | 55.7 ± 2.9 | 0.764 |
| LV GLS (%) | -19.2 ± 1.9 | -19.1 ± 2.6 | -18.8 ± 3.0 | 0.678 |
| **Right Ventricle** | | | | |
| RV EDVi (mL/m^2^) | 82.8 ± 12.8 | 85.7 ± 15.6 | 77.3 ± 14.1 | 0.073 |
| RV ESVi (mL/m^2^) | 37.1 ± 7.8**^#^** | 39.9 ± 9.9***^*^*^§^** | 34.2 ± 9.1**^#^** | 0.021 |
| RV SVi (mL/m^2^) | 45.7 ± 6.8 | 45.7 ± 7.4 | 43.1 ± 7.1 | 0.323 |
| RV EF (%) | 55.4 ± 4.7**^#^** | 53.7 ± 4.8***^*^*** | 56.1 ± 5.6 | 0.041 |
| RV GLS (%) | -22.2 ± 3.5**^#^** | -20.7 ± 3.0***^*^*** | -21.6 ± 4.2 | 0.021 |
| **Left ventricular myocardial work** | | | | |
| GWI (mmHg·%) | 2008.6 ± 337.7 | 1948.9 ± 341.0 | 1931.8 ± 317.0 | 0.393 |
| GCW (mmHg·%) | 2029.2 ± 343.1 | 2013.5 ± 400.7 | 2029.9 ± 284.5 | 0.956 |
| GWW (mmHg·%) | 62.2 ± 45.0**^#^** | 38.7 ± 33.2***^*^*** | 52.4 ± 35.4 | 0.001 |
| GWE (%) | 95.7 ± 4.7 | 97.3 ± 4.4 | 96.9 ± 3.7 | 0.061 |
| GWIV (mmHg∙%∙mL) | 10579.1 ± 3030.4 | 10035.8 ± 2760.6 | 10055.9 ± 2622.7 | 0.420 |
| GCWV (mmHg∙%∙mL) | 10744.0 ± 3076.5 | 10252.0 ± 2805.3 | 10518.3 ± 2824.8 | 0.554 |
| GWWV (mmHg∙%∙mL) | 249.6 ± 203.4**^#^** | 145.3 ± 133.8***^*^*** | 179.3 ± 114.1 | 0.003 |
| GWEV (%∙mL) | 96.5 ± 4.1**^#^** | 98.01 ± 3.4***^*^*** | 97.6 ± 3.1 | 0.046 |
| **Right ventricular myocardial work** | | | | |
| GWI (mmHg·%) | 552.9 ± 127.2 | 529.3 ± 115.7 | 500.7 ± 144.7 | 0.165 |
| GCW (mmHg·%) | 546.4 ± 126.8 | 514.9 ± 110.8 | 493.8 ± 139.9 | 0.093 |
| GWW (mmHg·%) | 20.8 ± 15.9 | 27.2 ± 21.9 | 18.3 ± 7.4 | 0.182 |
| GWE (%) | 96.1 ± 3.3**^#^** | 94.4 ± 5.2***^*^*** | 96.2 ± 1.5 | 0.011 |
| GWIV (mmHg∙%∙mL) | 3541.9 ± 1334.3**^§^** | 3342.8 ± 1237.8 | 2691.4 ± 1292.3***^*^*** | 0.033 |
| GCWV (mmHg∙%∙mL) | 3545.0 ± 1335.9**^§^** | 3326.8 ± 1206.6 | 2772.3 ± 1287.1***^*^*** | 0.037 |
| GWWV (mmHg∙%∙mL) | 84.9 ± 66.2 | 100.7 ± 74.8 | 104.7 ± 71.2 | 0.086 |
| GWEV (%∙mL) | 97.0 ± 3.1**^#^** | 95.6 ± 4.5***^*^*** | 96.1 ± 3.1 | 0.030 |

**Supplementary Table 6.** Comparison of the athlete population categorized by different sport disciplines

Continuous variables are presented as means ± SD; categorical variables are reported as frequencies (%). 3D, three-dimensional; EDVi, end-diastolic volume index; EF, ejection fraction; ESVi, end-systolic volume index; GCW, constructive myocardial work index; GCWV, volume-adjusted constructive myocardial work index; GLS, global longitudinal strain; GWE, myocardial work efficiency; GWEV, volume-adjusted myocardial work efficiency; GWI, global myocardial work index; GWIV, volume-adjusted global myocardial work index; GWW, wasted myocardial work index; GWWV, volume-adjusted wasted myocardial work index; LV, left ventricle; Mi, mass index; RV, right ventricle; SVi, stroke volume index; VO2/kg, peak oxygen uptake indexed to body weight.

*P < 0.05 vs. mixed group, #P < 0.05 vs. endurance group, §P < 0.05 vs. power group

**Supplementary Table 7. Basic correlations between demographic data, 3D LV and RV morphological and functional, myocardial work parameters and peak exercise capacity in athletes**

|  | **VO_2_/kg** | |
| --- | --- | --- |
|  | **r** | **p** |
| **Basic anthropometric and demographic data** | | |
| BSA (m^2^) | 0.0225 | 0.718 |
| Systolic blood pressure (mmHg) | 0.1286 | 0.038 |
| Diastolic blood pressure (mmHg) | -0.0426 | 0.494 |
| Heart rate (bpm) | -0.1301 | 0.036 |
| **Training-specific characteristics** | | |
| Competitive training since (years) | 0.0573 | 0.366 |
| Training time (h/week) | -0.0486 | 0.435 |
| Endurance sports class | 0.2649 | <0.001 |
| **Biventricular 3D Morphology and Function** | | |
| LV EDVi (mL/m2) | 0.3514 | <0.001 |
| LV ESVi (mL/m2) | 0.3620 | <0.001 |
| LV SVi (mL/m2) | 0.2373 | <0.001 |
| LV Mi (g/m2) | 0.3119 | <0.001 |
| LV EF (%) | -0.1992 | <0.001 |
| LV GLS (%) | 0.2662 | <0.001 |
| RV EDVi (mL/m2) | 0.3868 | <0.001 |
| RV ESVi (mL/m2) | 0.3899 | <0.001 |
| RV SVi (mL/m2) | 0.2836 | <0.001 |
| RV EF (%) | -0.2329 | <0.001 |
| RV GLS (%) | 0.0427 | 0.493 |
| **Left ventricular myocardial work** | | |
| GWI (mmHg·%) | -0.1501 | 0.015 |
| GCW (mmHg·%) | -0.1673 | 0.007 |
| GWW (mmHg·%) | 0.0421 | 0.499 |
| GWE (%) | -0.0797 | 0.200 |
| GWIV (mmHg∙%∙mL) | 0.1545 | 0.013 |
| GCWV (mmHg∙%∙mL) | 0.1463 | 0.018 |
| GWWV (mmHg∙%∙mL) | 0.1075 | 0.084 |
| GWEV (%∙mL) | -0.0983 | 0.114 |
| **Right ventricular myocardial work** | | |
| GWI (mmHg·%) | 0.1376 | 0.029 |
| GCW (mmHg·%) | 0.1284 | 0.041 |
| GWW (mmHg·%) | 0.2044 | 0.001 |
| GWE (%) | 0.0326 | 0.601 |
| GWIV (mmHg∙%∙mL) | 0.3024 | <0.001 |
| GCWV (mmHg∙%∙mL) | 0.3007 | <0.001 |
| GWWV (mmHg∙%∙mL) | 0.2341 | <0.001 |
| GWEV (%∙mL) | 0.0433 | 0.487 |

Continuous variables are presented as means ± SD; categorical variables are reported as frequencies (%). 3D, three-dimensional; EDVi, end-diastolic volume index; EF, ejection fraction; ESVi, end-systolic volume index; GCW, constructive myocardial work index; GCWV, volume-adjusted constructive myocardial work index; GLS, global longitudinal strain; GWE, myocardial work efficiency; GWEV, volume-adjusted myocardial work efficiency; GWI, global myocardial work index; GWIV, volume-adjusted global myocardial work index; GWW, wasted myocardial work index; GWWV, volume-adjusted wasted myocardial work index; LV, left ventricle; Mi, mass index; RV, right ventricle; SVi, stroke volume index.
